# An objective bone conduction verification tool using a piezoelectric thin-film force transducer

**DOI:** 10.3389/fnins.2022.1068682

**Published:** 2022-11-17

**Authors:** Yafei Nie, Jinqiu Sang, Chengshi Zheng, Jian Xu, Fangjie Zhang, Xiaodong Li

**Affiliations:** ^1^Key Laboratory of Noise and Vibration Research, Institute of Acoustics, Chinese Academy of Sciences, Beijing, China; ^2^University of Chinese Academy of Sciences, Beijing, China

**Keywords:** bone conduction hearing aids, audibility, objective measurements, verification, force level

## Abstract

All hearing aid fittings should be validated with appropriate outcome measurements, whereas there is a lack of well-designed objective verification methods for bone conduction (BC) hearing aids, compared to the real-ear measurement for air conduction hearing aids. This study aims to develop a new objective verification method for BC hearing aids by placing a piezoelectric thin-film force transducer between the BC transducer and the stimulation position. The newly proposed method was compared with the ear canal method and the artificial mastoid method through audibility estimation. The audibility estimation adopted the responses from the transducers that correspond to the individual BC hearing thresholds and three different input levels of pink noise. Twenty hearing-impaired (HI) subjects without prior experience with hearing aids were recruited for this study. The measurement and analysis results showed that the force transducer and ear canal methods almost yielded consistent results, while the artificial mastoid method exhibited significant differences from these two methods. The proposed force transducer method showed a lower noise level and was less affected by the sound field signal when compared with other methods. This indicates that it is promising to utilize a piezoelectric thin-film force transducer as an *in-situ* objective measurement method of BC stimulation.

## Introduction

Hearing is the sense by which we perceive the sounds around us, through hearing we interact with our environment and communicate with others. Hearing loss is one of the major public health problems, and the findings of the World Health Organization (WHO) indicated that more than 1.5 billion people currently have varying degrees of hearing loss, which could grow to 2.5 billion by 2050 (WHO, 2021). Hearing loss types can be categorized as conductive hearing loss, sensorineural hearing loss, and mixed hearing loss. Acoustic hearing aids are one of the most predominantly used hearing interventions (Kochkin, 2009). These devices are designed to restore the audibility of low-level sounds, maximize the intelligibility of conversational-level speech, and maintain comfort with loud sounds (Mueller, 2005; Dillon, 2012). There are three typical types of acoustic hearing aids: air conduction (AC) hearing aids, bone conduction (BC) hearing aids, and active middle ear hearing implants (AMEI) (Rahne and Plontke, 2022). The purpose of BC hearing aids is to assist in providing hearing rehabilitation for patients with conductive or mixed hearing loss or unilateral deafness who are unable to use conventional AC hearing aids (Ellsperman et al., 2021). BC hearing aids can be classified as conventional bone conduction devices (BCDs) and implantable BCDs, depending on whether surgical implantation is required. Currently available percutaneous BCDs include the Baha five and Baha six connect systems (Cochlear Ltd., Sydney, Australia) (Kim et al., 2017; Kruyt et al., 2020) and the Ponto three and Ponto four systems (Oticon A/S, Smorum, Denmark) (Lagerkvist et al., 2020); the available passive transcutaneous BCDs include the Baha five Attract (Cochlear Ltd., Sydney, Australia) (Oberlies et al., 2020) and Sophono Alpha2 (Medtronic, Dublin, Ireland) (Kajosaari et al., 2017; Willenborg et al., 2022); and the available active transcutaneous BCDs include the BONEBRIDGE (MED-EL, Innsbruck, Austria) (Sprinzl et al., 2021; Seiwerth et al., 2022) and the Osia (Cochlear Ltd., Sydney, Australia) (Goldstein et al., 2021; Willenborg et al., 2022). The conventional BCDs are coupled to the skin either by an adhesive ADHEAR (MED-EL, Innsbruck, Austria) (Dobrev et al., 2019; Zernotti et al., 2021) or by simple pressure [softband (Oticon A/S, Smorum, Denmark, Cochlear Ltd., Sydney, Australia) or Baha SoundArc (Cochlear Ltd., Sydney, Australia)] (Rahne and Plontke, 2022).

The amplification specified for each hearing aid user is based on the user’s hearing loss with an appropriate prescription formula, such as NAL-NL2 and DSL v5.0 (Scollie et al., 2005; Keidser et al., 2011). Validation methods are needed to verify whether the initial-fit settings are consistent with the prescription targets. The aided sound field thresholds have been used for many years to verify the audibility of AC and BC hearing aids (Hawkins, 2004). However, the aided sound field thresholds have some clear limitations that include poor test-retest reliability, susceptibility to the room and circuit noise, possibility of off-frequency listening, time-consuming nature of the measurements, provision of information only for low-level signals, and inaccuracies known to be associated with non-linear signal processing (Hawkins, 2004). The real-ear measurement replaced the aided sound field thresholds to verify the AC hearing aids (Hawkins et al., 1989; Stelmachowicz et al., 2002), a well-defined method for verifying that the real-ear output of the AC hearing aid matches the prescription target and can be used for further fine-tuning. Some studies have shown that the real-ear measurement approach significantly improves speech intelligibility in quiet environments when compared with the initial fitting (Boymans and Dreschler, 2012; Chang et al., 2018; Valente et al., 2018). However, the objective verification of the audibility provided by BC hearing aids for individual patients remains a challenge. Clinicians still often resort to using aided sound field thresholds to assess the fitting of the BC hearing aids, thus motivating us to develop *in-situ* objective measurement methods as for AC hearing aids.

Several researchers have attempted toward setting up real-ear measurement methods for BC hearing aids. Hodgetts et al. (2010) studied objective alternatives to aided sound field thresholds for BC hearing aids. They estimated the audibility of aided speech for Baha users using three different approaches: the aided sound field approach, the real-ear approach, and the accelerometer approach. Although the accelerometer approach was the most accurate approach, it was not practically feasible as the moving mass of the transducer was not accessible in the clinical products. Mertens et al. (2014) presented a study in which they placed a microphone in the occluded contralateral ear canal to measure the input-output functions of the BONEBRIDGE (MED-EL, Innsbruck, Austria). Hodgetts and Scollie (2017) proposed a method using a skull simulator that allows for a similar verification approach to a coupler-based SPL approach, which is only for percutaneous BCDs. Several previous studies have shown that there were inter-individual differences in the mastoid impedance, which would lead to differences in the output force of the BCDs (Flottorp and Solberg, 1976; Nie et al., 2022). Therefore, other types of BC hearing aids, such as ADHEAR, Ponto/Baha on a headband or softband, that vibrate through the intact skin cannot be measured accurately with current simulators (artificial mastoid or skull simulator). In addition, BC hearing aids with stimulation at the condyle are now available on the market, of which the position cannot match existing skull simulators. The difference in impedance between the mastoid and condyle is significant (Nie et al., 2022). Therefore the output from the current skull simulators cannot predict the output from the condyle. A skin microphone (SM) method has been developed with the objective of verifying the fitting of BCDs (Hodgetts et al., 2018; Persson et al., 2022). This method is based on a sound-insulated SM placed on the forehead as a sensor for bone vibration, which may be applied to all types of BCDs. However, this approach does not directly measure the vibration output of the BCDs. It is highly desirable to develop an objective verification method suitable for BC hearing aids that are coupled to intact skin, which can accurately measure the output of the BCDs at the stimulation position with the low noise level, little affected by the sound field signal and exerts little effects on the mechanical impedance of the stimulation position.

The primary aim of this study is to measure the audibility of a master BC hearing aid *in-situ*, on hearing-impaired subjects using a new method based on a piezoelectric thin-film force transducer, which measures the vibration force generated by BCDs at the stimulation position. The secondary aim is to investigate whether there are differences in the estimation of audibility between the new method and the other two methods (ear canal method and artificial mastoid method). The tertiary aim is to investigate the influence of noise level and the sound field signal on the objective measurement methods. Finally, the advantages and disadvantages of the three methods are discussed.

## Materials and methods

### Subjects

Twenty subjects, eight males, and twelve females participated in the experiments. They were between 49 and 68 years old with a mean age of 58.6 years. The average BC PTA_4_ (500, 1,000, 2,000, and 4,000 Hz) for all 20 subjects was 13.7 dB HL (SD = 5.9), and the average AC PTA_4_ (500, 1,000, 2,000 and, 4,000 Hz) for all 20 subjects was 27 dB HL (SD = 4.8). Eighteen subjects had mild conductive hearing loss, and two subjects had moderate mixed hearing loss. However, none of the subjects wore hearing aids.

### Apparatus

The experiments were conducted in a sound-treated booth. An audiometer (Conera Audiometer GN Otometrics Ltd., Denmark), a TDH-39 headphone (Melison, China), and a BC transducer B81 (RadioEar, Middelfart, Denmark) were used to measure the subjects’ AC and BC hearing thresholds. The master BC hearing aid used in the present study had one input channel and one output channel. For the input channel, an electret condenser omnidirectional microphone was used. For the output channel, the BC transducer B81 was used in the present study. The transducer B81 was mounted on the subject’s mastoid using RadioEar’s steel spring headband P-3333 to ensure a tight connection with the skin, and it is crucial that B81 does not touch the auricle during the measurements. The hearing aid function of the master BC hearing aid was implemented on a microprocessor platform STM32L476 (ARM, America) with a sampling frequency of 16 kHz, and the ADAU1777 (Analog Devices, America) was responsible for the analog to digital conversion, and the digital to analog conversion. In the present study, the hearing aid was programmed for a 20 dB HL at audiometric frequencies (250, 500, 1,000, 2,000, 3,000, 4,000, 6,000, and 8,000 Hz) using the NAL-R algorithm (Byrne and Dillon, 1986). The master BC hearing aid was a linear device without non-linear signal processing. Other features, such as feedback cancelation, noise reduction, and beamforming were not included in the master BC hearing aid processing.

The force transducer used in the present study was a master system comprising two primary elements: (1) a PVDF piezoelectric-film transducer SDT1-028K, (2) a charge amplifier VK102, which can provide four gain steps. The output of the master system was a voltage signal, of which the force transducer was calibrated using the artificial mastoid (B&K 4930, Brüel & Kjær, Denmark). The sensitivity of the force transducer was obtained at all audiometric frequencies (250, 500, 750, 1,000, 1,500, 2,000, 3,000, 4,000, 6,000, and 8000 Hz). In addition, the artificial mastoid (B&K 4930, Brüel & Kjær, Denmark) was also used to measure the force level (FL) generated by the master BC hearing aid in the present study.

The probe microphone of the ER-10C DPOAE Probe System (Etymotic Research, Elk Grove Village, Illinois, USA) was used to measure the sound pressure level (SPL) in the subject’s ear canal. The ER-10C probe microphone, equipped with a “regular” foam eartip (ER10C-14A, diameter 13 mm, Etymotic Research, Elk Grove Village, Illinois, USA), was cautiously inserted into the subject’s ear canal. A “baby” foam eartip (ER10C-14B, diameter 10 mm, Etymotic Research, Elk Grove Village, Illinois, USA) was used for two subjects with narrow ear canals.

A multi-channel signal analyzer (B&K-3560-C, Brüel & Kjær, Denmark) was used for signal acquisition and analysis. The generator section of the analyzer was connected to a loudspeaker (Genelec 8030C, Finland) to generate the sound field signals. A standard microphone (B&K 4189, Brüel & Kjær, Denmark) was connected to the analyzer’s input channel to monitor the sound field signals on line. The force transducer signals, probe microphone signals, and B&K 4930 artificial mastoid signals were all measured using the signal analyzer. All measured signals were calculated using the analyzer’s FFT with a frequency range of 0.1–10 kHz. An average of 100 measurements was adopted to improve the signal-to-noise ratio, requiring a total measurement time of approximately 5 s.

### Experimental procedures

#### Measurements of the hearing thresholds and the aided responses

Two quantities are needed for the estimation of audibility: the value corresponding to the BC hearing threshold *HL*_*f*_ and the value corresponding to the aided response *Audibility*_*f,input*_. This section describes how to measure these two quantities.

The BC pure-tone hearing thresholds for each subject were measured at 250, 500, 750, 1,000, 1,500, 2,000, 3,000, 4,000, 6,000, and 8,000 Hz with an audiometer (Conera Audiometer GN Otometrics Ltd., Denmark) and a B81 transducer. The B81 was coupled to the mastoid by RadioEar’s steel spring headband P-3333. The BC hearing thresholds were acquired at ten audiometry frequencies using the modified Hughson-Westlake procedure (Harrell, 2002). A mark was left on the subject’s skin for the B81 to be placed in the same position for subsequent measurements. This ensured that the position of the B81 remained fixed during the whole measurement for each subject.

**FIGURE 1:**
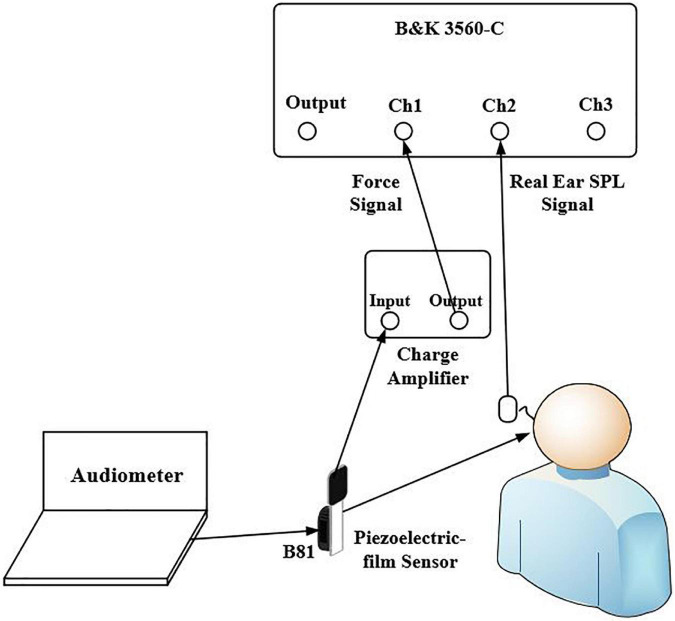
The experimental setup to determine the HL-to-SPL transform and the HL-to-FL (force transducer) transform. The B81 transducer was connected to the audiometer to output a signal at 30 dB HL per frequency. The probe microphone was placed in the ear canal for the SPL measurements and the force transducer was placed tightly between the B81 transducer and the stimulation position for the FL_FT measurements.

After the BC hearing thresholds were measured, the B81 was removed, and the subject rested for 5 min. The force transducer was then placed on the marked position for the FL measurements, and the probe microphone was placed in the ear canal for the SPL measurements. Finally, the B81 was placed tightly on the top of the force transducer and connected to the audiometer to transmit the signal. The experimental setup to determine the HL-to-SPL transform and the HL-to-FL transform (force transducer) for each subject is shown in Figure 1. The audiometer dial was set to 30 dB HL to ensure a steady signal that consistently exceeded the noise level of the measurement system. The audiometer and the B81 provided signals at each audiometric frequency, while the signal analyzer (B&K-3560-C) simultaneously acquired signals from the force transducer and the probe microphone. The process of obtaining the HL-to-FL transform (artificial mastoid) was similar to the other two transforms, the output force was measured by the artificial mastoid. By combining the above transform methods with the measured BC hearing thresholds of the subject, the FL_FT, the SPL, and the FL_AM corresponding to the subject’s BC hearing threshold could be obtained. The FL_FT and FL_AM represent the force level measured by the force transducer method and artificial mastoid method, respectively. For example, if the FL_FT value for 1,000 Hz at 30 dB HL on the audiometer dial for a given subject was 63.7 dB FL, and the actual BC hearing threshold value for that subject was 15 dB HL, the true *in-situ* FL_FT for that subject at BC hearing threshold would be 48.7 dB FL [63.7 dB–(30 dB–15 dB)]. The SPL and FL_AM at BC hearing thresholds were estimated in the same way with the assumption of linearity. The reason for using the above transform method to indirectly obtain the FL and SPL at the BC hearing threshold is that in some cases, the FL and SPL at the BC hearing threshold cannot be measured directly due to the noise.

**FIGURE 2:**
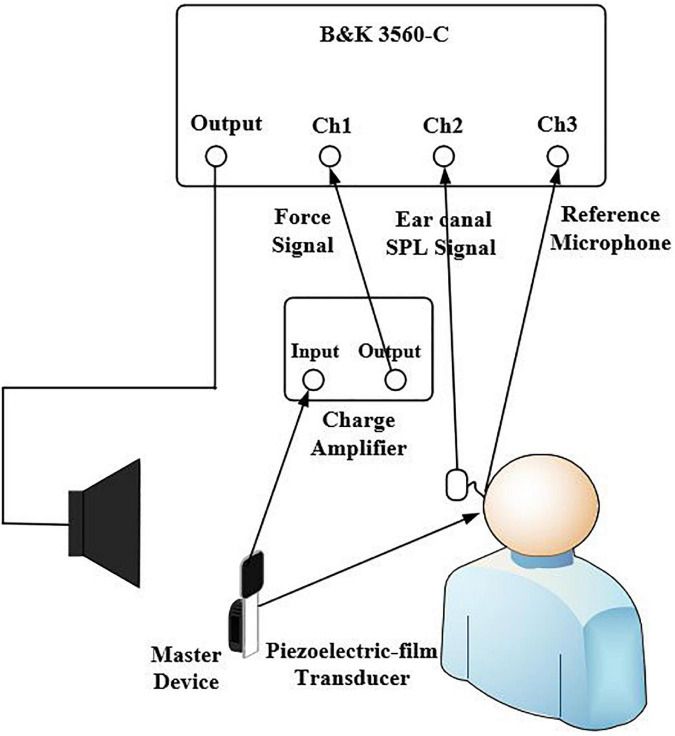
Experimental setup for the force transducer and the probe microphone aided response measurements. The loudspeaker was used to present the pink noise and the reference microphone was placed at the entrance to the master BC hearing aids microphone to monitor the overall SPL of the signal. The probe microphone was placed in the ear canal for the SPL measurements and the force transducer was placed tightly between the B81 transducer and the stimulation position for the FL_FT measurements.

**FIGURE 3:**
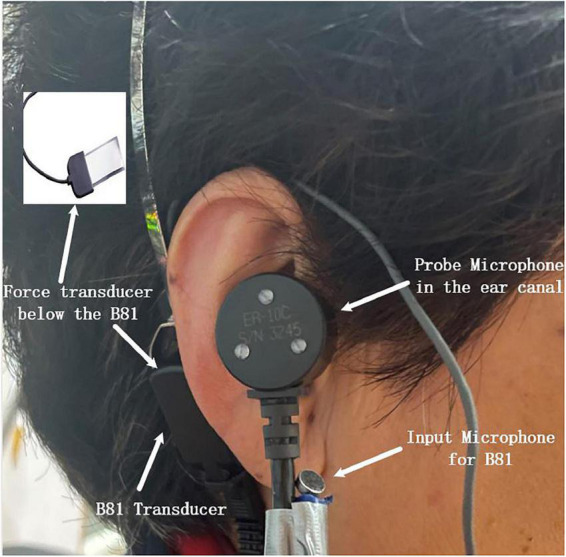
The master bone conduction (BC) hearing aid was connected to a subject during the aided response measurements. The input microphone and B81 transducer were the input and the output of the master BC hearing aid, respectively. The probe microphone in the ear canal was used for the SPL measurements, and the force transducer under the B81 transducer (tightly between the B81 transducer and the mastoid) was used for the FL_FT measurements. The small picture in the upper left corner shows the piezoelectric thin-film force transducer.

The experimental setup for aided response measurements is shown in Figure 2. The picture of the experimental setup for the aided response measurements of one subject is presented in Figure 3. To avoid acoustic feedback, the input and output of the master BC hearing aid were separately working during the present measurement. The hearing aid first recorded the signal through the input microphone, amplified the signal, and then played the aided signal through the BC transducer B81. It should be noted that the loudspeaker only worked when the hearing aid was recording the signal and was muted when the hearing aid was playing the aided signal through the BC transducer. The audibility was ultimately determined by the aided response measurements. The subject was seated in the center of room 1.4 m from a loudspeaker mounted 1.26 m from the floor in the corner of the room. The subject wore the force transducer, the master BC hearing aid, and the probe microphone in turn. Pink noise signals were delivered from the loudspeaker with an overall SPL of 55, 65, and 75 dB SPL, respectively. A reference microphone B&K 4930 was placed close to the master BC hearing aid microphone to monitor the SPL of the pink noise. The FL_FT response and the ear canal SPL response of the master BC hearing aid for each input were recorded simultaneously and analyzed by the signal analyzer (B&K-3560-C). Finally, the loudspeaker was turned off, and the noise level of the force transducer and the probe microphone was measured when the master BC hearing aid was turned on and off, respectively. For the FL_AM measurements with the B&K 4930 artificial mastoid, the BC transducer of the hearing aid was placed on the B&K 4930 artificial mastoid, and the other settings remain the same. The probe microphone, the force transducer, and the master BC hearing aid were removed from the subject and repositioned to determine the measurement repeatability of both methods. Similarly, measurements on the B&K 4930 artificial mastoid were repeated twice. Note that the position of the BC transducer was kept as consistent as possible in both measurements.

#### Measurement of the sound field signal effect

In order to avoid feedback, in the aided response measurement described in section “Measurements of the hearing thresholds and the aided responses”, the input and the output processes of the master BC hearing aid were separated in time. Although it was not consistent with the actual operating state of the hearing aid, this measurement can mimic the *in-situ* signal level from the BC stimulation for the three objective methods. To investigate whether the ear canal method and the force transducer method would be affected by the sound field signal from the loudspeaker when measuring the aided response in clinical application, the following experiments were performed. First, when the hearing aid was turned off and the loudspeaker played pink noise at 55, 65, and 75 dB SPL alternatively, the measured sound field effects on the probe microphone and the force transducer were recorded; Then, when the BC transducer of the hearing aid played aided pink noise and the loudspeaker replayed pink noise simultaneously at each stimulation level (55, 65, and 75 dB SPL), the measured BC stimulation responses plus the sound field effects on the probe microphone and the force transducer were recorded.

### Audibility estimation

The audibility estimated by the force transducer method was determined by subtracting the force levels corresponding to the subject’s BC hearing thresholds from the force transducer measured aided response at each stimulation level (55, 65, and 75 dB SPL). The audibility calculation can be expressed by the following equation:


$$A\,u\,d\,i\,b\,i\,l\,i\,t\,y_{f,i\,n\,p\,u\,t}=A\,i\,d\,L\,e\,v\,e\,l_{f,i\,n\,p\,u\,t}-H\,L_{f}.$$

Where, *Audibility*_*f*,*input*_ indicates the audibility at the frequency *f* with input (55, 65, 75 dB SPL), *AidLevel*_*f*,*input*_ indicates the aided response at the frequency *f* with input (55, 65, 75 dB SPL), and *HL*_*f*_ indicates the magnitude corresponding to the subject’s BC hearing threshold at the frequency *f*. For example, if the FL_FT at 1,000 Hz at 55 dB SPL input for a given subject was 70 dB FL, and the FL_FT of the BC hearing threshold at 1,000 Hz for that subject was 48.7 dB FL, the audibility would be 21.3 dB FL (70 dB–48.7 dB). The audibility estimated with the ear canal method and the artificial mastoid method was calculated in the same way as for the force transducer method. For the artificial mastoid method, there were no inter-individual differences in the force levels measured by the artificial mastoid under the same stimulus input. In addition, it needs to note that amplitude was used to calculate the force level.

### Statistical analysis

A 3 (transducer) × 3 (input level) × 10 (frequency) repeated measures ANOVA was used to test significant main effects and interactions for audibility. Then the paired-samples *t*-tests were used to make planned comparisons of interest. The Bonferroni correction was used to minimize type 1 error associated with multiple comparisons. In total, 90 comparisons were performed: 3 transducers, 3 input levels, and 10 frequencies. This resulted in a corrected *p*-value of 0.00056. The intraclass correlation coefficients (ICCs) were used to test the consistency of the three methods for two repeated measurements.

## Results

### The results of the estimated audibility

**FIGURE 4:**
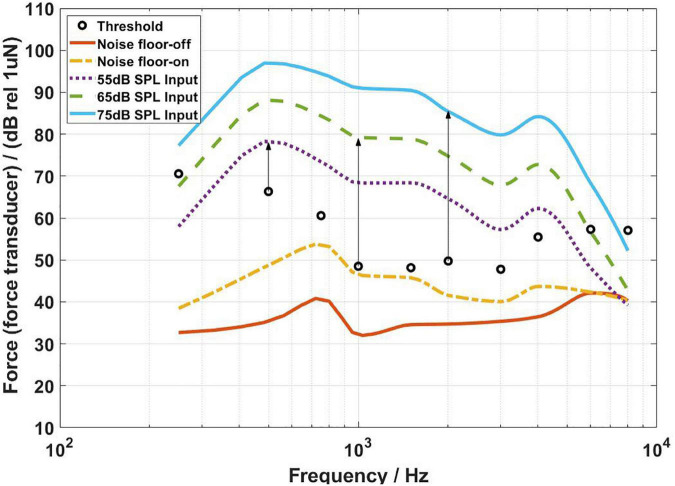
The FL_FT corresponding to the bone conduction (BC) hearing threshold (referenced to the B81 transducer stimulation position) and the aided responses at 55, 65, and 75 dB SPL input levels. The arrows depict the audibility calculations.

**FIGURE 5:**
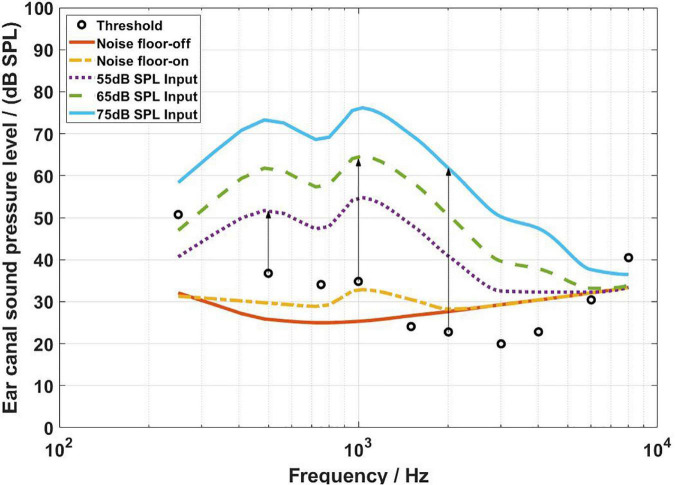
The sound pressure level (SPL) corresponding to the bone conduction (BC) hearing threshold (referenced to the ear canal SPL) and the aided responses at 55, 65, and 75 dB SPL input levels. The arrows depict the audibility calculations.

**FIGURE 6:**
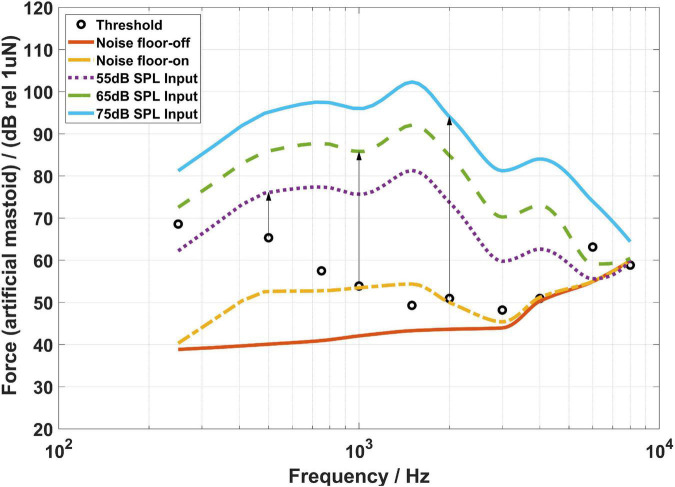
The FL_AM corresponding to the bone conduction (BC) hearing threshold (referenced to the artificial mastoid) and the aided responses at 55, 65, and 75 dB SPL input levels. The arrows depict the audibility calculations.

Figures 4–6 show the BC hearing thresholds and the measured aided responses at 55, 65, and 75 dB SPL input levels averaged across all 20 subjects for the force transducer, ear canal, and artificial mastoid methods, respectively. The black circles in Figures 4–6 represent the corresponding mean values of FL_FT, SPL, and FL_AM for the 20 subjects at their BC hearing thresholds, respectively. The “Noise level-off” indicates the noise level of the measurement system with the hearing aid turned off and the loudspeaker turned off. The “Noise level-on” indicates the noise level of the measurement system with the hearing aid turned on and the loudspeaker turned off. From Figures 4–6 it can be observed that there are differences between the “Noise level-off” and the “Noise level-on”. The noise level measured when the BC hearing aid turned off can be assumed to come from the transducer’s inherent noise. The noise level measured when the hearing aid turned on may be a combination of the circuit noise of the master BC hearing aid, the inherent noise of the transducer, and the room background noise amplified by the hearing aid. As shown in Figure 4, except for 6,000 and 8,000 Hz, the noise level of the force transducer and the master BC hearing aid is much lower than the aided responses. Figure 5 shows that the noise level of the probe microphone and the master BC hearing aid is much lower than the aided responses below 2,000 Hz, and the aided responses are close to the noise level at 6,000 and 8,000 Hz for 55 and 65 dB SPL input levels. Figure 6 shows that the noise level of the artificial mastoid and the master BC hearing aid is much lower than the aided responses, except for 6,000 and 8,000 Hz at low input levels.

As shown in Figure 4, all threshold levels and the aided response levels (except for 55 dB SPL input level at 8,000 Hz) are above the noise level of the force transducer. The reason is that the force transducer is placed below the BC transducer, allowing direct measurement of the output force of the BC transducer, and the force transducer has a low noise level. However, Figure 5 shows that the threshold levels in the range of 1,500–6,000 Hz are below the noise level of the probe microphone. The ear canal method measures the SPL in the ear canal due to BC vibrations through the probe microphone. It was found that the ear canal SPL corresponding to 30 dB HL in the range of 1,500–6,000 Hz for each subject was small and close to the noise level of the probe microphone during the measurements, thus resulting in the ear canal SPL that corresponds to the mean BC hearing thresholds below the noise level. However, the mean value of the subject’s ear canal SPL corresponding to 30 dB HL at 8,000 Hz was approximately 60 dB SPL, therefore the ear canal SPL corresponding to the BC hearing threshold level at 8,000 Hz in Figure 5 is above the noise level of the probe microphone. The artificial mastoid method is similar to the force transducer method, which directly measures the output force of the BC transducer, with a slightly higher noise level compared to the force transducer, so the mean BC hearing thresholds at most frequencies correspond to the force levels interfered with the noise level of the artificial mastoid. In addition, a similar phenomenon can be observed in Figures 4–6, where the aided response at 8,000 Hz for 55 dB SPL input interferes with the noise level of the transducer. This is due to the fact that when measuring the aided response corresponding to the 55 dB SPL input level, the input microphone of the master BC hearing aid receives a very small signal at 8,000 Hz, which is below the noise level of the input microphone. It can also be seen from Figure 5 that the aided response at 6,000 Hz for 55 dB SPL input level and 6,000 and 8,000 Hz for 65 dB SPL input level all interfere with the noise level of the probe microphone. Firstly, because the small vibration force of the B81 leads to a lower aided response at high frequencies, and secondly, because at high frequencies, the ear canal SPL caused by the BC is also smaller than that at low frequencies. Similarly, Figure 6 shows that the aided responses at 6,000 Hz and 8,000 Hz for 55 dB SPL input level and 8,000 Hz for 65 dB SPL input level interfere with the noise level of the artificial mastoid, which may be caused by the insufficient high-frequency vibration of the B81 and relatively high noise level of the artificial mastoid. The resonance peak at 4,000 Hz in Figure 4 and the resonance peaks at 1,500 and 4,000 Hz in Figure 6 correspond to the characteristics of the output force of the B81 transducer.

**FIGURE 7:**
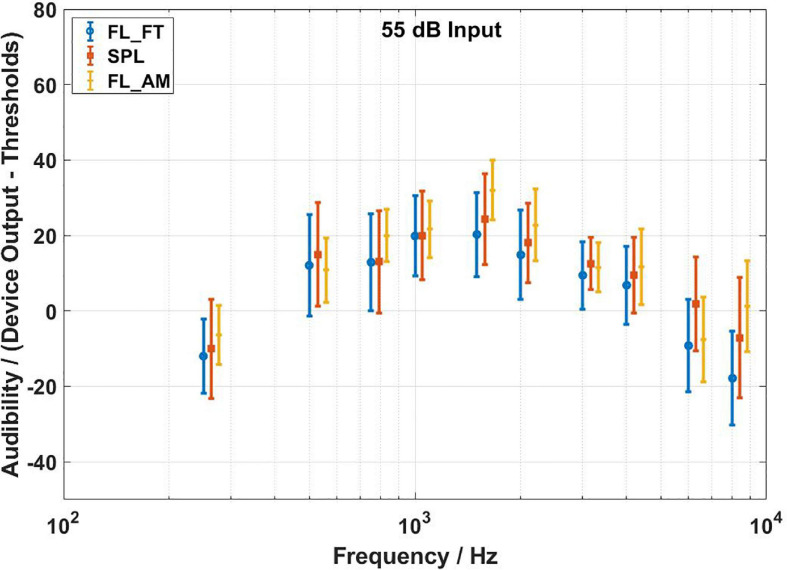
Audibility estimates for the force transducer method, the ear canal method, and the artificial mastoid method at 55 dB SPL input level. Average values over all subjects. Error bars indicate ± 1 SD. The FL_FT indicates the force transducer method, the SPL indicates the ear canal method, and the FL_AM indicates the artificial mastoid method.

**FIGURE 8:**
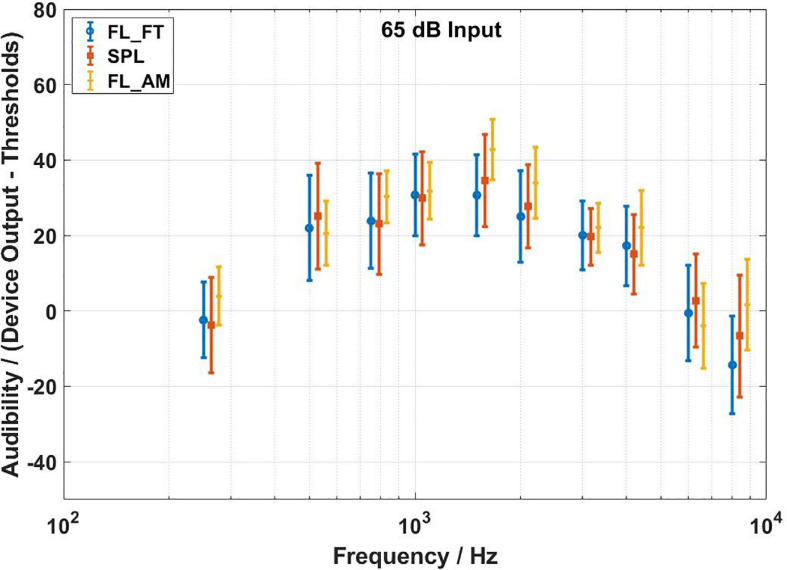
Audibility estimates for the force transducer method, the ear canal method, and the artificial mastoid method at 65 dB SPL input level. Average values over all subjects. Error bars indicate ± 1 SD. The FL_FT indicates the force transducer method, the SPL indicates the ear canal method, and the FL_AM indicates the artificial mastoid method.

**FIGURE 9:**
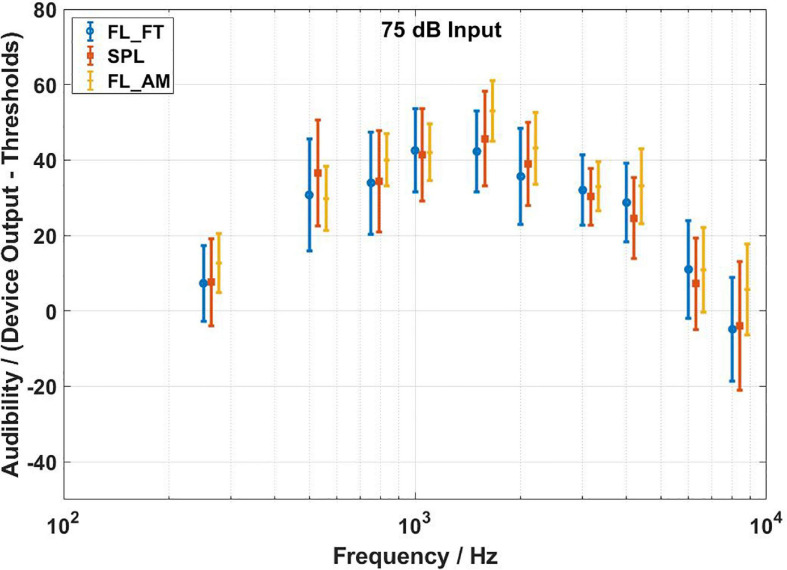
Audibility estimates for the force transducer method, the ear canal method, and the artificial mastoid method at 75 dB SPL input level. Average values over all subjects. Error bars indicate ± 1 SD. The FL_FT indicates the force transducer method, the SPL indicates the ear canal method, and the FL_AM indicates the artificial mastoid method.

The audibility estimates results of the three methods are shown in Figures 7–9 for input levels of 55, 65, and 75 dB SPL, respectively. The plots show average values over all subjects, and the error bars indicate 1 SD. The arrows at 500, 1,000, and 2,000 Hz in Figures 4–6 indicate the audibility calculations. A 3 (transducer) × 3 (input level) × 10 (frequency) repeated measures ANOVA revealed a significant three-way interaction [*F*(36,684) = 28.717,*p* < 0.0001], so the paired-samples *t*-tests were used to proceed with the planned comparisons of interest. Tables 1–3 shows the mean differences for all 90 contrasts of interest. Cohen’s d was used to estimate the effect size.

**TABLE 1:** Results from the *t*-test showing the *p*-values when comparing the audibility in 55 dB SPL input for the differences between the three measuring methods (FL_FT_ represents the force transducer method, SPL represents the ear canal method, and FT_AM_ represents the artificial mastoid method). The calculations of effect size are based on (Cohen, 1992). Large effect sizes of 0.8 or greater are highlighted in grey.

Input level	Planned comparison		Mean1-Mean2	Pooled SD	Effect size	*t*	*p*
55 dB Input	250	FL_FT_ -SPL	–1.937	11.715	0.165	–0.739	0.469
	250	FL_FT_ - FL_AM_	–5.617	5.978	0.940	–4.202	0.000
	250	SPL - FL_AM_	–3.680	10.590	0.348	–1.554	0.137
	500	FL_FT_ - SPL	–2.906	10.943	0.266	–1.188	0.25
	500	FL_FT_ - FL_AM_	1.286	9.836	0.131	0.585	0.566
	500	SPL - FL_AM_	4.193	9.709	0.432	1.931	0.069
	750	FL_FT_ - SPL	–0.190	8.712	0.022	–0.098	0.923
	750	FL_FT_ - FL_AM_	–7.048	9.696	0.727	–3.251	0.004
	750	SPL - FL_AM_	–6.858	8.773	0.782	–3.663	0.002
	1,000	FL_FT_ - SPL	–0.146	8.780	0.017	–0.074	0.942
	1,000	FL_FT_ - FL_AM_	–1.829	7.001	0.261	–1.169	0.257
	1,000	SPL - FL_AM_	–1.684	5.920	0.284	–1.272	0.219
	1,500	FL_FT_ - SPL	–4.006	8.383	0.478	–2.137	0.046
	1,500	FL_FT_ - FL_AM_	–11.770	6.409	1.836	–8.212	0.000
	1,500	SPL - FL_AM_	–7.764	6.787	1.144	–5.116	0.000
	2,000	FL_FT_ - SPL	–3.185	6.504	0.490	–2.19	0.041
	2,000	FL_FT_ - FL_AM_	–7.938	6.406	1.239	–5.542	0.000
	2,000	SPL - FL_AM_	–4.753	4.058	1.171	–5.238	0.000
	3,000	FL_FT_ - SPL	–3.120	6.712	0.465	–2.079	0.051
	3,000	FL_FT_ - FL_AM_	–2.110	5.455	0.387	–1.73	0.1
	3,000	SPL - FL_AM_	1.010	3.877	0.261	1.165	0.258
	4,000	FL_FT_ - SPL	–2.650	6.376	0.416	–1.859	0.079
	4,000	FL_FT_ - FL_AM_	–4.892	4.667	1.048	–4.687	0.000
	4,000	SPL - FL_AM_	–2.242	4.491	0.499	–2.232	0.038
	6,000	FL_FT_ - SPL	–11.020	6.580	1.675	–7.489	0.000
	6,000	FL_FT_ - FL_AM_	–1.671	3.417	0.489	–2.187	0.041
	6,000	SPL - FL_AM_	9.349	6.569	1.423	6.364	0.000
	8,000	FL_FT_ - SPL	–10.712	6.261	1.711	–7.651	0.000
	8,000	FL_FT_ - FL_AM_	–19.091	3.676	5.193	–23.224	0.000
	8,000	SPL - FL_AM_	–8.379	5.731	1.462	–6.538	0.000

**TABLE 2:** Results from the *t*-test showing the *p*-values when comparing the audibility in 65 dB SPL input for the differences between the three measuring methods (FL_FT_ represents the force transducer method, SPL represents the ear canal method, and FT_AM_ represents the artificial mastoid method). The calculations of effect size are based on (Cohen, 1992). Large effect sizes of 0.8 or greater are highlighted in grey.

Input level	Planned comparison		Mean1-Mean2	Pooled SD	Effect size	*t*	*p*
65 dB Input	250	FL_FT_ - SPL	1.379	10.213	0.135	0.604	0.553
	250	FL_FT_ - FL_AM_	–6.351	6.141	1.034	–4.625	0.000
	250	SPL - FL_AM_	–7.729	8.946	0.864	–3.864	0.000
	500	FL_FT_ - SPL	–3.191	11.756	0.271	–1.214	0.24
	500	FL_FT_ - FL_AM_	1.337	10.267	0.130	0.582	0.567
	500	SPL - FL_AM_	4.528	10.014	0.452	2.022	0.057
	750	FL_FT_ - SPL	0.781	7.989	0.098	0.437	0.667
	750	FL_FT_ - FL_AM_	–6.410	9.357	0.685	–3.064	0.006
	750	SPL - FL_AM_	–7.191	8.596	0.836	–3.741	0.000
	1,000	FL_FT_ - SPL	0.824	8.810	0.094	0.418	0.68
	1,000	FL_FT_ - FL_AM_	–1.114	6.990	0.159	–0.713	0.485
	1,000	SPL - FL_AM_	–1.938	6.248	0.310	–1.387	0.181
	1,500	FL_FT_ - SPL	–3.865	8.020	0.482	–2.155	0.044
	1,500	FL_FT_ - FL_AM_	–12.107	5.767	2.099	–9.388	0.000
	1,500	SPL - FL_AM_	–8.242	6.944	1.187	–5.308	0.000
	2,000	FL_FT_ - SPL	–2.731	6.510	0.419	–1.876	0.076
	2,000	FL_FT_ - FL_AM_	–8.881	6.384	1.391	–6.222	0.000
	2,000	SPL - FL_AM_	–6.150	4.597	1.338	–5.982	0.000
	3,000	FL_FT_ - SPL	0.381	6.915	0.055	0.247	0.808
	3,000	FL_FT_ - FL_AM_	–2.012	5.923	0.340	–1.519	0.145
	3,000	SPL - FL_AM_	–2.393	3.416	0.701	–3.133	0.005
	4,000	FL_FT_ - SPL	2.282	6.736	0.339	1.515	0.146
	4,000	FL_FT_ - FL_AM_	–4.768	4.778	0.998	–4.463	0.000
	4,000	SPL - FL_AM_	–7.050	5.883	1.198	–5.359	0.000
	6,000	FL_FT_ - SPL	–3.326	6.693	0.497	–2.222	0.039
	6,000	FL_FT_ - FL_AM_	3.428	4.072	0.842	3.766	0.000
	6,000	SPL - FL_AM_	6.754	6.059	1.115	4.986	0.000
	8,000	FL_FT_ - SPL	–7.706	5.969	1.291	–5.774	0.000
	8,000	FL_FT_ - FL_AM_	–15.932	4.306	3.700	–16.547	0.000
	8,000	SPL - FL_AM_	–8.226	5.832	1.411	–6.308	0.000

**TABLE 3:** Results from the *t*-test showing the *p*-values when comparing the audibility in 75 dB SPL input for the differences between the three measuring methods (FL_FT_ represents the force transducer method, SPL represents the ear canal method, and FT_AM_ represents the artificial mastoid method). The calculations of effect size are based on (Cohen, 1992). Large effect sizes of 0.8 or greater are highlighted in grey.

Input level	Planned comparison		Mean1-Mean2	Pooled SD	Effect size	*t*	*p*
75 dB Input	250	FL_FT_ -SPL	–0.377	8.737	0.043	–0.193	0.849
	250	FL_FT_ - FL_AM_	–5.366	6.038	0.889	–3.974	0.000
	250	SPL - FL_AM_	–4.989	8.271	0.603	–2.697	0.014
	500	FL_FT_ - SPL	–5.848	11.886	0.492	–2.200	0.040
	500	FL_FT_ - FL_AM_	0.920	10.912	0.084	0.377	0.710
	500	SPL - FL_AM_	6.768	10.278	0.658	2.945	0.008
	750	FL_FT_ - SPL	–0.395	7.823	0.050	–0.226	0.824
	750	FL_FT_ - FL_AM_	–6.128	10.038	0.610	–2.73	0.013
	750	SPL - FL_AM_	–5.733	8.649	0.663	–2.964	0.008
	1,000	FL_FT_ - SPL	1.128	9.624	0.117	0.524	0.606
	1,000	FL_FT_ - FL_AM_	0.497	7.893	0.063	0.282	0.781
	1,000	SPL - FL_AM_	–0.631	6.123	0.103	–0.461	0.650
	1,500	FL_FT_ - SPL	–3.365	7.994	0.421	–1.883	0.075
	1,500	FL_FT_ - FL_AM_	–10.708	5.770	1.856	–8.299	0.000
	1,500	SPL - FL_AM_	–7.343	7.054	1.041	–4.655	0.000
	2,000	FL_FT_ - SPL	–3.317	7.052	0.470	–2.104	0.049
	2,000	FL_FT_ - FL_AM_	–7.456	6.608	1.128	–5.046	0.000
	2,000	SPL - FL_AM_	–4.139	4.532	0.913	–4.084	0.000
	3,000	FL_FT_ - SPL	1.778	7.444	0.239	1.068	0.299
	3,000	FL_FT_ - FL_AM_	–1.009	6.218	0.162	–0.725	0.477
	3,000	SPL - FL_AM_	–2.787	3.503	0.795	–3.891	0.001
	4,000	FL_FT_ - SPL	4.102	7.480	0.548	2.453	0.024
	4,000	FL_FT_ - FL_AM_	–4.353	4.634	0.939	–4.201	0.000
	4,000	SPL - FL_AM_	–8.456	6.587	1.284	–5.741	0.000
	6,000	FL_FT_ - SPL	3.829	6.172	0.620	2.774	0.012
	6,000	FL_FT_ - FL_AM_	0.180	4.288	0.042	0.187	0.853
	6,000	SPL - FL_AM_	–3.649	5.443	0.670	–2.998	0.007
	8,000	FL_FT_ - SPL	–0.838	6.569	0.128	–0.57	0.575
	8,000	FL_FT_ - FL_AM_	–10.439	5.369	1.944	–8.695	0.000
	8,000	SPL - FL_AM_	–9.601	7.097	1.353	–6.05	0.000

There were 16 comparisons between the force transducer method and the artificial mastoid method that yielded significant differences in the estimation of audibility. fifteen pairs of them mainly occurred at 250, 1,500, 2,000, 4,000, and 8,000 Hz for each input level, with the audibility estimated by the artificial mastoid method significantly higher than that by the force transducer method. The remaining pair was at 6,000 Hz for the 65 dB SPL input with the audibility estimated by the artificial mastoid method significantly lower than that by the force transducer method. fifteen comparisons yielded significant differences in the estimation of audibility between the ear canal method and the artificial mastoid method. Thirteen pairs of them showed higher audibility by the artificial mastoid method than that by the ear canal method, mainly occurring at 1,500, 2,000, and 8,000 Hz for all the three input levels, and at 250, 750, and 4,000 Hz for the 65 dB SPL input level, and 4,000 Hz for the 75 dB SPL input level. The remaining two pairs occurred at 6,000 Hz for the 55 and 65 dB SPL input levels, with the audibility estimated by the artificial mastoid method significantly lower than that by the ear canal method.

Three comparisons between the force transducer and ear canal methods yield significant differences in the audibility estimates, at 6,000 and 8,000 Hz for 55 dB SPL input level, and 8,000 Hz for 65 dB SPL input level. All three pairs of the above significantly different comparisons show that lower audibility is estimated by the force transducer method than the ear canal method. In addition, ICCs were calculated for two repeated measurements for each subject, and the results showed good agreement for repeated measurements for all three methods. The average ICC values of the three methods at the individual level were 0.958 (force transducer method, range: 0.873–0.993), 0.957 (ear canal method, range: 0.85–0.996), and 0.986 (artificial mastoid method, range: 0.969–0.992).

### The effect of the sound field signal

**FIGURE 10:**
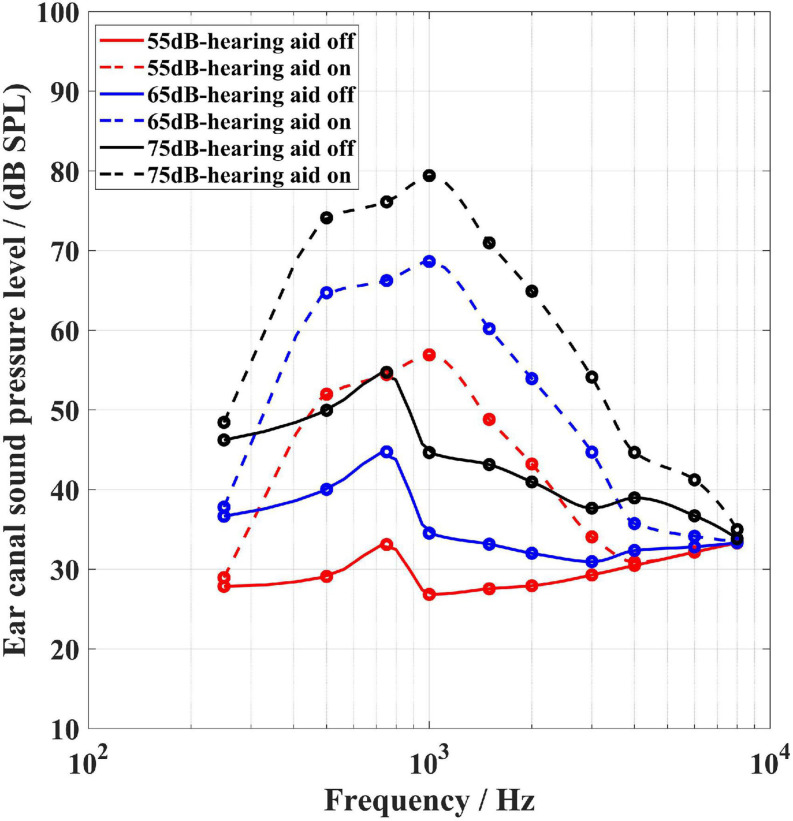
Results of the ear canal sound pressure level (SPL) measurement in one subject. The solid lines show the ear canal SPL is measured by the probe microphone with the loudspeaker turned on and the BC hearing aid turned off, while the dashed lines indicate the ear canal SPL is measured by the probe microphone with the loudspeaker turned on and the BC transducer of the hearing aid playing the aided signal. The red, blue, and black lines indicate the stimulation inputs with an overall SPL of 55, 65, and 75 dB, respectively.

**FIGURE 11:**
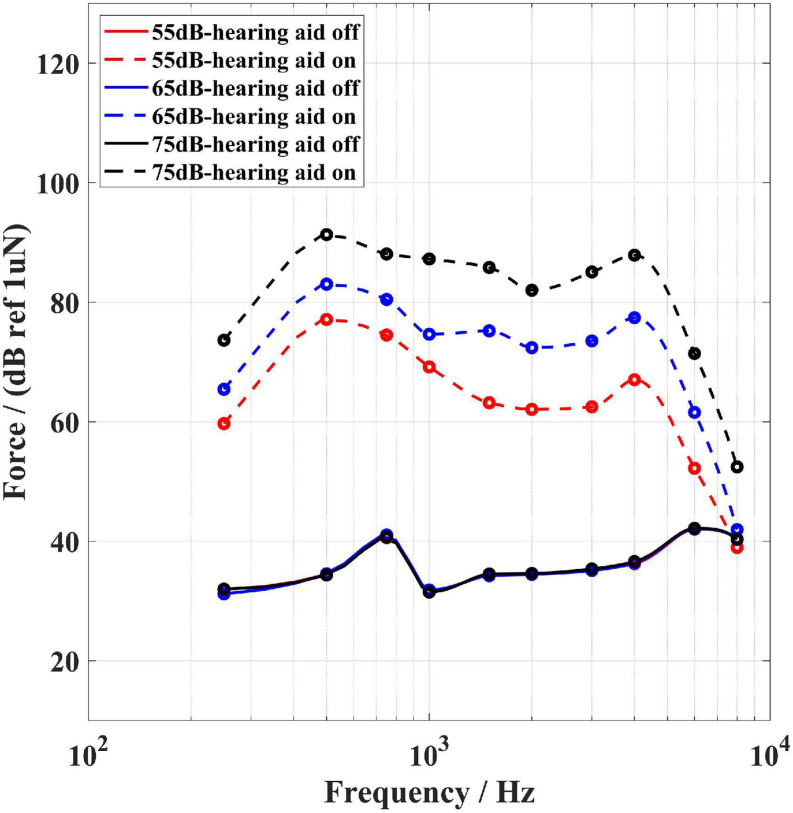
Results of the force level measurement in one subject. The solid lines show that the FL is measured by the force transducer with the loudspeaker turned on and the BC hearing aid turned off, while the dashed lines indicate the force level is measured by the force transducer with the loudspeaker turned on and the BC transducer of the hearing aid playing the aided signal. The red, blue, and black lines indicate the stimulation inputs with an overall SPL of 55, 65, and 75 dB, respectively. The three solid lines were equal.

Figures 10, 11 indicate the responses measured by the probe microphone and the force transducer, respectively. The solid lines show the responses measured with the hearing aid turned off and the loudspeaker turned on, and the dashed lines show responses measured with both the hearing aid and the loudspeaker turned on. The red, blue, and black lines show the signal measured when the loudspeaker was playing pink noise at 55, 65, and 75 dB SPL, respectively. From Figure 10 it can be found that the SPLs in the ear canal at 250 Hz are relatively close in both cases (solid line and dashed line) at each stimulation level. It indicates that the SPL in the ear canal with the hearing aid turned on is mainly from the sound field signal by the loudspeaker. In this case, the measurements at 250 Hz must be considered invalid since most of the measured aided responses do not come from bone conduction. It can be concluded that in clinical use the ear canal method may be influenced by the sound field signal from the loudspeaker. It should be noted that this problem did not exist in the present audibility results. Because in the formal aided response measurements, the input and output processes of the hearing aid were separated to avoid acoustic feedback, and the loudspeaker was muted when the BC transducer of the hearing aid played the aided signal. In Figure 11, the three solid lines are equal and far below the three dashed lines (except for 8,000 Hz). As can be seen in Figure 4, the three solid lines in Figure 11 indicate the noise level of the force transducer measurement system. The results indicate that the force transducer is not influenced by the sound field signal when measuring the aided responses.

### The effect of the force transducer on the impedance of the stimulation position

The output properties of BCDs are affected by the mechanical impedance of the load (Weece and Allen, 2010). Several researchers have thoroughly investigated the impedance of the mastoid, and the results showed that the mastoid impedance was influenced by age (Flottorp and Solberg, 1976; Zhang, 1994; Mackey et al., 2016; Nie et al., 2022)), and that differences in mastoid impedance will lead to differences in the output force of the BC transducer. In addition, there are some differences in mastoid impedance between Chinese subjects and B&K 4930 artificial mastoid (Nie et al., 2022). Accurate measurement of the output generated by BCDs at each subject’s stimulation position requires that the measuring tool does not affect the mechanical impedance of the stimulation position. Whether the force transducer proposed in the present study can meet the above requirement was tested.

**FIGURE 12:**
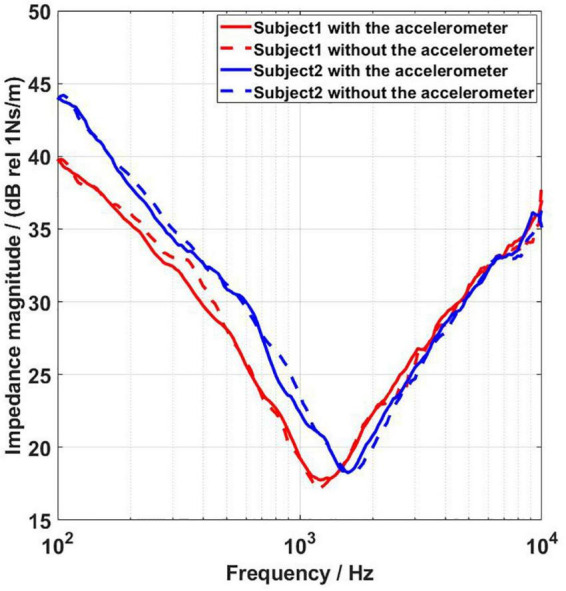
Results of mastoid impedance measurements in two subjects. Subject 1 was a 60-year-old female, and subject 2 was a 24-year-old male. The dashed lines show the subject’s mastoid impedance, and the solid lines show the mechanical impedance measured when the force transducer is attached to the mastoid.

Using the mechanical impedance measurement method in the study of (Nie et al., 2022), the mastoid impedances of two subjects were measured as well as the mechanical impedance of the mastoid when the force transducer was attached to the mastoid. The experimental results are shown in Figure 12. The dashed line shows the subject’s mastoid impedance and the solid line shows the mechanical impedance measured when the force transducer was attached to the mastoid. Subject 1 and subject 2 were a 61-year-old female and a 24-year-old male, respectively. Figure 12 shows that the presence of the force transducer does not change the mechanical impedance of the mastoid. Such a force transducer can also be used to measure the output of BCDs with stimulation at other locations, such as condyle.

## Discussion

### A comparison of the three methods

We developed and tested a new *in-situ* method (one force transducer) and then compared this new method with the ear canal method and the artificial mastoid method. Since all three methods use the same hearing aid to estimate the same target (the audibility), equivalent results would have indicated that there is no absolute superiority or inferiority between the three methods. However, the analysis results show that there are significant differences between the three methods. From the analysis results, it can be seen that only three comparisons between the force transducer and ear canal methods show significant differences. It can be inferred that the force transducer and ear canal methods almost yielded equivalent results. Both the force transducer and ear canal methods were significantly different from the artificial mastoid method, suggesting that the force transducer and ear canal methods estimate audibility more accurately. The advantages and limitations of each of the three methods are analyzed below.

#### The characteristics of the ear canal method

The ear canal method has some advantages. This method does not require special equipment or calibration and is more similar to the clinician fitting the AC hearing aids. However, BC hearing aid users with atresia, chronic drainage or ear wax problems should not use this method as it may block the probe microphone. In the experiment described in section “Measurement of the sound field signal effect”, it was found that despite the presence of foam earplugs, at the low frequency of 250 Hz, the sound field signal that entered the ear canal appeared to introduce a comparative level as the BC stimulation signal in the probe microphone. We conducted measurements on one subject, to compare the ear canal SPL between two conditions, of which one was measured with the hearing aid turned off and the loudspeaker turned on, and the other was measured with the hearing aid turned on and the loudspeaker turned on. It was found that the SPLs in the ear canal at 250 Hz were essentially equal in both conditions (see Figure 10), indicating that the SPLs in the ear canal with the hearing aid turned on were mainly from the sound field signal. Therefore the measurements at 250 Hz must be considered invalid since most of the measured aided response does not come from bone conduction. This finding is consistent with the study by (Hodgetts et al., 2010). This problem may be related to two factors. First, at low frequencies, the earplugs are not well insulated and the microphone in the ear canal picks up the sound field signal; second, it is related to the amplification characteristics of the hearing aids, most of which provide a relatively low output at 250 Hz. However, during the aided response measurements in the present study, the loudspeaker was muted when the output of the hearing aid was working, so the responses measured by the probe microphone were not affected by the sound field signal from the loudspeaker during the aided response measurements. Therefore, the audibility results measured by the ear canal method at 250 Hz were valid in the present study. But such limitation by the ear canal method remains in practical hearing aids, where the input and output always work simultaneously. In addition, (Hodgetts et al., 2010) presents another limitation of the ear canal method, namely that most clinical probe microphone equipment have higher noise level at high frequencies relative to low frequencies, making it difficult to measure SPLs below about 40 dB SPL (Scollie and Seewald, 2002). In the present study, a low noise level, more sensitive microphone was adopted with a maximum noise level of 33 dB SPL. As shown in Figure 5, the noise level of the probe microphone still interfered with the aided responses of the hearing aid at 6,000 and 8,000 Hz for the 55 and 65 dB SPL input levels, which would make the audibility estimates at these frequencies inaccurate. This phenomenon is caused by several reasons. Firstly, the pink noise was used as the sound field signal in this study, which causes a low level sound field at high frequencies. When measuring the aided response corresponding to the 55 dB SPL input level, the input microphone of the hearing aid receives a small signal at 8,000 Hz, which is below the noise level of the input microphone, so the aided response interferes with the noise level. Secondly, the vibration force of the B81 is insufficient at high frequencies, resulting in a relatively small aided response at high frequencies. This is the reason why there were significant differences in audibility estimates between the force transducer and ear canal methods at 6,000 and 8,000 Hz for the 55 dB SPL input level and 8,000 Hz for the 65 dB SPL input level. Another point to note is that the ear canal method requires the use of a foam earplug, which may create an occlusion effect at low frequencies for some subjects (Stenfelt and Goode, 2005; Reinfeldt et al., 2007; Surendran and Stenfelt, 2021). Theoretically, in both the hearing threshold measurements and the aided response measurements, the foam earplugs were not removed and the estimated audibility may not be sensitive to the occlusion effect.

In addition to using the sound pressure in the ear canal to estimate BC sound, there are other methods of sound pressure measurement. The nasal sound pressure (NSP) was used to verify the bone conduction implant’s functionality during and after surgery (Reinfeldt et al., 2019). In their preclinical study, they found that the NSP gave higher signal-to-noise than the ear canal sound pressure from the same stimulation. A restriction in their results is that the noise level of the microphone keeps the NSP valid only for a limited frequency range of 0.4–5 kHz. Lately, a new, so-called, surface microphone method has been developed with the objective of verifying the fitting of BCDs (Persson et al., 2022). This method has some advantages over the NSP method, since it does not require the patients to hold their breath during the measurement and is easier to apply to patients. This method suffers from the same problem as the ear canal method, in that at high frequencies, the aided response is limited by the noise level of the measurement system. Compared with the ear canal method, the NSP method and SM method can be used for patients with atresia or chronic drainage.

#### The characteristics of the artificial mastoid method

The artificial mastoid method has some limitations compared to the other two methods. First, compare with the force transducer and ear canal methods, this method requires special equipment, B&K 4930 artificial mastoid. Second, this method does not take into account the differences between subjects and the simulator artificial mastoid, which can lead to discrepancies between the results measured by the method and the true results. There were significant differences between artificial mastoid impedance and Chinese mastoid impedance, and there were also significant inter-individual differences in the mastoid impedance of the subjects (Nie et al., 2022). The variability of the mechanical impedance affects the output of the BC hearing aids (Nie et al., 2022).

#### The characteristics of the force transducer method

**FIGURE 13:**
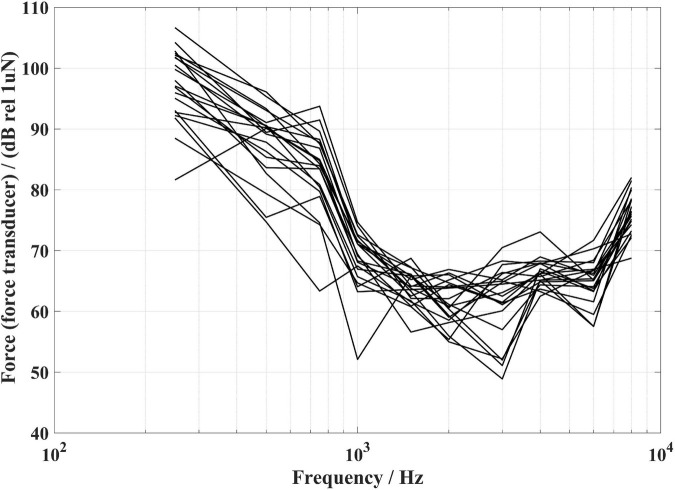
The FL_FT corresponding to a fixed 30 dB HL bone conduction (BC) at each frequency for the 20 subjects.

The force transducer method used in the present study has significant advantages over the other two methods. Compared with the ear canal method, the force transducer method directly measures the vibration force generated by the BC transducer at the stimulation position, and the noise level of the force transducer is relatively low, therefore the high frequencies signals at low input levels can be acquired during the aided response measurements, so the estimated audibility at high frequencies is more accurate. This method can be used for BC hearing aid users with canal atresia or chronic drainage. In addition, the results of the experiments described in section “Measurement of the sound field signal effect” show that the signal collected by the force transducer does not contain the sound field signal during the aided response measurements (see Figure 11). It can be concluded that the signal measured by the force transducer during the aided response measurement is only the vibration signal of the BC hearing aid at the stimulation position excluding the sound field interference. In contrast to the artificial mastoid method, the force transducer method can measure the vibration response of the BC hearing aid at the stimulation position of each subject, taking into account the output differences of BC hearing aid caused by impedance differences between individuals. Figure 13 represents the FL_FT corresponding to a fixed 30 dB HL (BC) stimulation at each frequency for the 20 subjects. It can be seen that even though the input voltages of the BC transducer are the same, the differences in mastoid impedance between subjects lead to differences in the BC force level on each subject. It can be deduced that even though the subjects’ BC hearing thresholds are equal, there will be differences in the corresponding BC force values, whereas the artificial mastoid method cannot show such individual differences. In addition, the force transducer in the present study can also monitor the output of the BC headphones at other stimulation positions such as the condyle, but the artificial mastoid is not suitable due to the significant differences between the human condyle impedance and the artificial mastoid impedance (Nie et al., 2022).

### The limitations of the present study

The present study still has some limitations. One limitation is that real speech was not used in the aided response measurements. As the measurement time may last for a long time when collecting the real speech, it is inevitable to include subjects’ breathing and movement in the measurement process, which will contaminate the measured results. Therefore, pink noise was chosen which can be collected in a short time (1–2 s) to avoid the above problems. Another limitation is that the force transducer method was currently only validated for headband-worn BCDs. The applicability such as using a piezoelectric thin-film force transducer tightly pressed by a headband to measure the skull vibration from the percutaneous BC hearing aids or the fully implantable BC hearing aids needs to be further investigated.

## Conclusion

Compared with the AC hearing aids, the objective verification method of audibility for BC hearing aid users has not been well established until now. In the present study, we proposed an objective verification method suitable for non-surgical bone conduction hearing aids by using a piezoelectric thin-film force transducer, which can measure the force level at the stimulation position directly. Experiments were conducted to estimate audibility in 20 hearing-impaired subjects using the master BC hearing aid. The measurement and analysis results showed that the proposed force transducer method and the ear canal method produced almost equal audibility estimation results, and both were significantly different from the artificial mastoid method. The force transducer method has some advantages, as it can be used for patients suffering from atresia or chronic drainage, without changing the mechanical impedance of the measured position, with the low noise level, and less affected by the sound field signal. The outcome of the present study suggests that the force transducer method can be used as an objective verification method for non-surgical BC hearing aids. In addition, it also can be used to monitor the output of BCDs at other positions such as the condyle which is usually stimulated by BC headphones.

## Data availability statement

The original contributions presented in this study are included in the article/supplementary material, further inquiries can be directed to the corresponding author.

## Ethics statement

The studies involving human participants were reviewed and approved by the Ethics Committee of the Institute of Acoustics Chinese Academy of Sciences. The patients/participants provided their written informed consent to participate in this study.

## Author contributions

YN: measurement, writing—original draft and review, and editing. CZ: writing—review and editing. JS: methodology and writing—review and editing. JX: hardware. FZ: hardware. XL: supervision and writing—review and editing. All authors contributed to the article and approved the submitted version.
